# Teacher emotional support and learning engagement: a sequential mediation model of basic psychological needs satisfaction and enjoyment

**DOI:** 10.3389/fpsyg.2026.1795248

**Published:** 2026-03-26

**Authors:** Xiaolan Ye, Kun He

**Affiliations:** 1 School of Foreign Languages, Qiongtai Normal University, Haikou, Hainan, China; 2 School of Physical Education, Qiongtai Normal University, Haikou, Hainan, China

**Keywords:** academic emotion, academic engagement, BPNs, chain mediation, foreign language enjoyment, perceived teacher support

## Abstract

**Objectives:**

Based on Self-Determination Theory, this study explores the mechanism by which teacher emotional support (TES) influences learning engagement of English as a foreign language (EFL) students, focusing on the mediation roles of basic psychological needs (BPNs) satisfaction and foreign language enjoyment.

**Methods:**

A cross-sectional investigation was conducted with 442 EFL undergraduates in Hainan Province. Data were collected using validated scales measuring teacher emotional support, basic psychological needs satisfaction, foreign language enjoyment, and learning engagement. Path analysis and bootstrap analysis were conducted to test the hypothesized sequential mediation model.

**Results:**

Findings revealed teacher emotional support significantly positively predicted learning engagement (β = 0.258) and operated through three indirect pathways: (1) the primary mediating role of basic psychological needs satisfaction (β = 0.211); (2) the modest mediating role of foreign language enjoyment (Effect = 0.061); (3) the sequential mediating role of basic needs satisfaction and foreign language enjoyment (Effect = 0.048). The total indirect effect size is 0.319 and the total effect is 0.577.

**Conclusion:**

Teacher emotional support enhances EFL students' learning engagement through multiple pathways. The most potent of these is the satisfaction of basic psychological needs. Foreign language enjoyment also serves as a significant mediator. Furthermore, a sequential chain wherein need satisfaction fosters enjoyment, which in turn drives engagement, was empirically supported. This research underscores the importance of addressing both motivational and emotional channels to optimize EFL classroom instruction.

## 1 Introduction

In recent years, learning engagement has drawn growing attention, largely because it is closely tied to positive educational outcomes. These outcomes include, but are not limited to, alleviation of academic boredom and dissatisfaction (Fredricks et al., 2004), higher academic aspirations and improved mental health (Archambault et al., 2009), higher school completion-rates (Reschly and Christenson, 2012), growth of learning efficiency and achievement (Dörnyei, 2019; Mercer, 2019), even as well as meaningful learning (Hiver et al., 2021a,b). Yet despite its importance, students actually become more and more disengaged as they progress through schools (Fredricks, 2011; Wang and Holcombe, 2010). Competing for students' attention and sustain their deep and continuous learning engagement against endless sources of distractions is a persistent challenge for educators and researchers worldwide (Mercer and Dörnyei, 2020). This challenge is particularly acute in English as a foreign language contexts, where learners must navigate not only cognitive demands but also the emotional turbulence inherent in mastering a new language—anxiety about making mistakes, fear of evaluation, and the frustration of limited self-expression (Dewaele and MacIntyre, 2014). In order to help educators better enhance students' learning engagement, researchers have identified a number of learner-internal factors (e.g., academic resilience) and learner-external factors (e.g., classroom environment) as important antecedents of EFL students' learning engagement. Notably, more and more studies have reported that the support provided by teachers is a core driver (e.g., Roorda et al., 2011; Havik and Westergård, 2019; Vargas-Madriz et al., 2024).

Among various forms of teacher support, teacher emotional support, characterized by care, respect, understanding of students, and responsiveness to students‘ emotional needs, stands out as an especially important factor influencing learning engagement (Ruzek et al., 2016; Fredricks et al., 2019; Granziera et al., 2022). When students perceive emotional support from their teachers, their learning engagement at behavioral, emotional, and cognitive levels significantly increases (Xu et al., 2023; Zhang et al., 2024). It is crucial to understand the underlying psychological mechanisms through which this process occurs (Guo et al., 2025). Grounded in Self-Determination Theory (Ryan and Deci, 2020), researchers have argued that support promotes engagement by satisfying students' basic psychological needs for autonomy, competence, and relatedness. Studies in technology-enhanced language learning have confirmed the mediating role of needs satisfaction (Li and Chiu, 2025). Complementing this perspective, Control-Value Theory (Pekrun, 2024) posits that positive emotions like enjoyment arise from appraisals of control and value and subsequently drive engagement. The mediating role of foreign language enjoyment has been well documented in EFL settings (Sadoughi and Hejazi, 2021; Liu and Zhou, 2024; Zhao and Yang, 2022). Emerging evidence further points to a sequential process wherein need satisfaction creates the psychological foundation for enjoyment, which in turn fuels engagement (Ma and Li, 2026).

Obviously, reseach gaps still persist. Most existing researches have examined the influence of teachers' emotional support on learning engagement in primary and secondary education, while its specific mechanisms for boosting learning engagement among college students remain under-explored (Ma et al., 2023). However, compared to K-12 students, university students may have greater autonomy, different motivational drivers, and more complex social-emotional dynamics with instructors, which might alter the mechanisms through which TES operates. Moreover, due to the distinctive interpersonal and social nature of EFL classrooms, teacher emotional support is particularly paramount for EFL students, because supportive teachers create a safe environment that reduces anxiety and encourages the risk-taking essential for language communication.

Based on the outlined introduction, the purpose of this study is to test a sequential mediation model in which basic psychological needs satisfaction and foreign language enjoyment serve as mediators in the relationship between teacher emotional support and learning engagement among EFL university students.

## 2 Theoretical framework and research hypotheses

### 2.1 Learning engagement

In a broad sense, engagement can be understood as a form of action (Lawson and Lawson, 2013). Action represents the defining and central feature of learning engagement (Skinner et al., 2009; Fredricks et al., 2016; Mercer, 2019). For example, Skinner et al. (2009) defined engagement as “energized, directed, and sustained actions”, and Reeve (2012) defined it as “the extent of a student's active involvement in a learning activity”. In a narrow sense, however, there is no consensus on the definition of learning engagement, including its subtypes and specific meanings, except that it is widely agreed that it is a multidimensional construct (Reschly and Christenson, 2012). Most commonly, learning engagement has been understood as encompassing three or four interrelated aspects (Fredricks et al., 2004, 2016; Wang and Fredricks, 2014). For example, Philp and Duchesne (2016) defined it as “a state of heightened attention and involvement, in which participation is reflected in cognitive, social, behavioral and affective dimensions as well”. Affective/emotional engagement relates to students' emotional attachment to school, including students' levels of interest, happiness, enjoyment, boredom, anxiety and feelings of belonging, identification and relatedness to teachers, peers and the school overall (Lawson and Lawson, 2013). Behavioral engagement refers to the amount and quality of students' participation in class and time spent on tasks (Reschly and Christenson, 2012). Cognitive engagement refers to sustained attention and mental effort in learning process (Philp and Duchesne, 2016). Social engagement denotes the quality and amount of interactions with and participation between interlocutors, characterizing the relational nature of language learning (Zhou et al., 2021).

In addition to these dimensions, recent scholarship has introduced agentic engagement as another key component (Reeve and Tseng, 2011; Reeve and Jang, 2022). Defined as students‘ proactive efforts to shape, enrich, and tailor their own learning experiences (Reeve and Jang, 2022), agentic engagement highlights the learner's active and constructive role in the instructional process. Unlike the prior dimensions, which focus largely on how students respond to given educational tasks, agentic engagement emphasizes students' capacity to influence and improve the conditions of their own learning. Learning engagement is shaped by a combination of contextual influences, such as teacher support, and internal personal factors, such as self-efficacy (Granziera et al., 2022; Yang et al., 2021).

### 2.2 The impact of teacher emotional support on EFL students' learning engagement

Teacher emotional support refers to students' perception of teacher warmth, care, liking, respect, as well as teachers' commitment to understanding students' thoughts, feelings, and behaviors (Pianta and Hamre, 2009; Roorda et al., 2017; Romano et al., 2020). This support is manifested through both one-on-one interactions and whole-class communication (Hughes, 2012). Building on the Classroom Assessment Scoring System (CLASS) framework developed by Pianta and colleagues (Pianta and Hamre, 2009), teacher emotional support is commonly conceptualized as encompassing three interrelated dimensions: positive climate, which reflects the overall emotional tone and connection between teachers and students; teacher sensitivity, which involves teachers' responsiveness to students' academic and emotional needs; and regard for adolescent perspective, which captures the extent to which teachers value and encourage students' autonomy, ideas, and social interactions (Romano et al., 2020; Schenke et al., 2015). These three dimensions collectively capture the multifaceted nature of emotional support that students experience in the classroom. And have been identified as particularly influential in students' academic development (Federici and Skaalvik, 2014).

The theoretical grounding for the link between teacher emotional support and student engagement draws from two complementary frameworks. Self-Determination Theory maintains that learning environments addressing students‘ basic psychological needs—autonomy, competence, and relatedness—are conducive to intrinsic motivation and sustained engagement (Ryan and Deci, 2020). Teaching behaviors that are emotionally supportive, such as expressing genuine concern for students, offering respectful feedback, and valuing students' ideas, directly nurture these needs (Reeve, 2016; Ahn et al., 2021; Pitzer and Skinner, 2017). Control-Value Theory complements this perspective by suggesting that contextual factors shape engagement through the emotions they elicit during learning (Pekrun, 2024). Teacher emotional support fosters a positive, encouraging, and effortful learning environment (Li et al., 2021; Granziera et al., 2022), which in turn enhances students‘ positive learning emotions such as enjoyment and hope (Xie and Guo, 2023). Together, these theoretical frameworks converge on a central premise: teacher emotional support serves as a critical resource that enhances students' willingness to invest effort and persist in learning tasks.

A substantial body of empirical evidence has substantiated this positive association across diverse EFL learning contexts. Meta-analytic evidence synthesizing 141 studies confirms that teacher support positively predicts school engagement, with emotional support emerging as a particularly robust predictor (Vargas-Madriz et al., 2024). In traditional classroom settings, perceived teacher support predicts behavioral, emotional, and cognitive engagement among EFL learners (Sadoughi and Hejazi, 2021), with both academic and emotional support contributing to learning engagement through mechanisms such as self-efficacy and achievement goal orientation (Liu et al., 2023). Longitudinal evidence further reveals reciprocal dynamics between teacher emotional support and emotional engagement: teacher support promotes engagement, while higher engagement simultaneously enhances learners' perception of teacher support over time (Alrabai et al., 2025). The positive role of teacher emotional support extends across various learning modalities, including online and technology-mediated environments (Fan, 2024; Sun et al., 2025; Quan et al., 2025; Zhao et al., 2026), suggesting that the human element of teaching remains indispensable regardless of the instructional medium.

The message from both theory and evidence is clear: when teachers provide emotional support, students are more likely to engage actively in learning. However, the mechanisms through which teacher emotional support exerts its influence remain largely under-explored in EFL higher education. Accordingly, as a foundation for investigating deeper pathways, we first propose:

*H1: Teacher emotional support is positively related to EFL students' learning engagement*.

### 2.3 The mediating role of basic psychological needs satisfaction

Building upon the Self-Determination Theory framework, this study examines the mediating role of basic psychological needs satisfaction. SDT posits three universal needs—autonomy, competence, and relatedness—whose fulfillment is crucial for motivation and engagement (Ryan and Deci, 2020). In EFL contexts, competence involves effectively enhancing language skills; relatedness entails feeling accepted by teachers and peers; and autonomy refers to self-endorsed regulation of learning activities (Ryan and Deci, 2020). These needs serve as the pathways through which supportive environments influence engagement (Zhen et al., 2017; Vasconcellos et al., 2020).

Teacher emotional support is uniquely positioned to satisfy each need. Such support nurtures relatedness by conveying warmth and respect (Ahn et al., 2021); fosters autonomy by respecting student perspectives and minimizing control (Reeve, 2016); and enhances competence through constructive feedback (Pitzer and Skinner, 2017). When these needs are satisfied, students experience greater intrinsic motivation, which translates into heightened engagement. Thus, basic psychological needs satisfaction is theorized to transmit the positive effect of teacher support to learning engagement. Therefore, grounded in SDT we propose:

*H2: Basic psychological needs satisfaction mediates the relationship between teacher emotional support and EFL learning engagement*.

### 2.4 The mediating role of foreign language enjoyment

Recent years have witnessed a growing emphasis on positive academic emotions in educational psychology, with enjoyment being paramount (Dewaele and Li, 2021). In second language acquisition, enjoyment—the feelings of pleasure, interest, and excitement toward learning activities—has gained recognition as a key positive emotion that promotes learning motivation and outcomes (Dewaele and MacIntyre, 2014).

Control-Value Theory offers a useful lens for understanding how teacher support cultivates this positive emotion. According to CVT, achievement emotions are elicited by students' cognitive appraisals of control and value (Pekrun, 2024). Teacher emotional support shapes both appraisals: when teachers express genuine concern and respect students' ideas, they create a psychologically safe environment that enhances perceived control over learning tasks; simultaneously, such support conveys that learning activities are worthwhile, increasing students' positive valuation of these activities. These dual appraisals directly elicit foreign language enjoyment, positioning emotionally supportive teachers as instrumental in cultivating positive emotions (Philp and Duchesne, 2016).

Foreign language enjoyment, in turn, promotes engagement through certain mechanisms. According to broaden-and-build theory, positive emotions broaden individuals' cognitive and motivational resources, fostering exploration, effort, and persistence (Fredrickson, 2001). The mediating role of enjoyment between teacher support and engagement has received consistent empirical support across EFL contexts (Sadoughi and Hejazi, 2021; Zhao and Yang, 2022; Li et al., 2025). These studies collectively confirm that teacher support enhances engagement largely through heightened enjoyment. However, what remains less explored is whether this emotional pathway operates independently or in concert with other psychological mechanisms—particularly the satisfaction of basic psychological needs. Therefore, we propose:

*H3: Foreign language enjoyment mediates the relationship between teacher emotional support and learning engagement*.

### 2.5 The sequential mediating role of basic psychological needs satisfaction and foreign language enjoyment

Another question that remains is whether the two mediators themselves are connected. Self-Determination Theory and Control-Value Theory together suggest they are: basic psychological needs satisfaction may serve as an antecedent to foreign language enjoyment. In other words, need satisfaction creates the psychological conditions that make enjoyment possible. Enjoyment, in turn, sustains engagement. Theoretically, a sequential pathway emerges.

Yet empirical validation of this specific sequence in EFL contexts remains limited. Although each individual association in the model has received empirical support, the integrated sequential chain has not been fully verified within the specific domain of EFL teaching. Alrabai and Algazzaz (2024) provided experimental evidence for a similar pathway, but their research focused only on emotional engagement, leaving the question of whether this chain extends to the multidimensional construct of learning engagement largely unanswered. Likewise, many existing studies have concentrated on autonomy support or teacher support in a broad sense, while emotional support as an independent prerequisite factor still requires targeted investigation (Liu et al., 2023). Most critically, research simultaneously examining basic psychological needs satisfaction and foreign language enjoyment as sequential mediating variables in a single model remains scarce in higher education EFL contexts. The majority of studies analyze these variables in isolation or as parallel mediators, overlooking the theoretically grounded causal sequential pathway.

To address these gaps, this study proposes a sequential mediation model. We hypothesize:

*H4: Basic psychological needs satisfaction and foreign language enjoyment sequentially mediate the relationship between teacher emotional support and learning engagement*.

**Figure 1:**
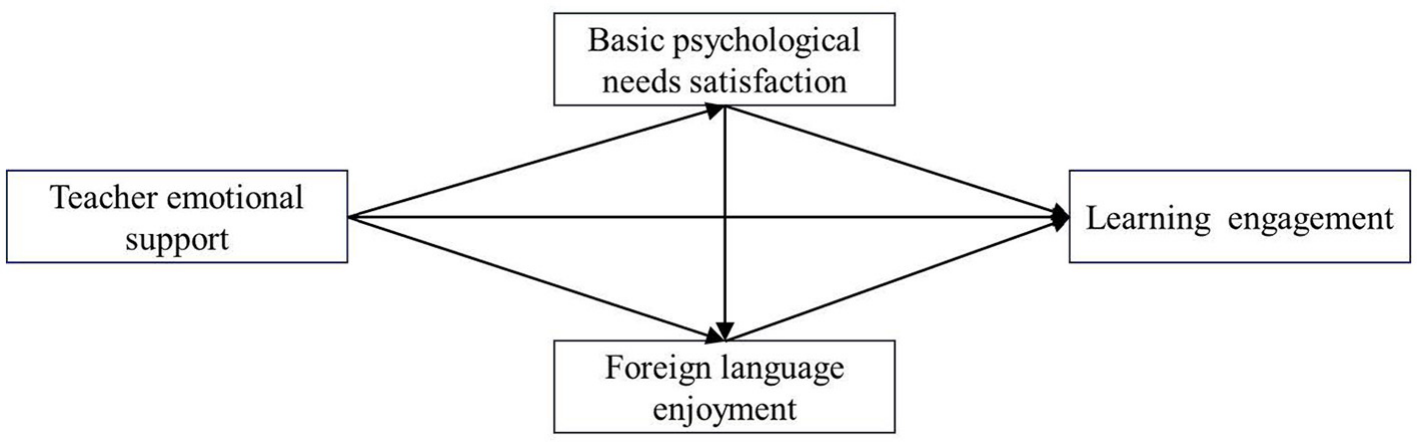
Conceptual model.

The conceptual model is detailed in Figure 1. By empirically validating this chain of mechanisms, this research can more accurately and comprehensively clarify the psychological path through which teacher emotional support is internalized by students. This research provides valuable theoretical inspiration and practical guidance for optimizing EFL teaching.

## 3 Research methodology

### 3.1 Participants

This study utilized a cross-sectional approach. The participants were non-English major freshmen and sophomores enrolled in universities across Hainan Province. These specific grade levels were selected because College English courses at universities in Hainan Province, just like in almost all the other provinces in China, are offered only during the 1st 2years of college. The sampling process was conducted in two steps. First, three universities were selected from a total of 21 institutions of higher education in the province using a convenience sampling strategy. This non-probability method was employed based on the principles of accessibility, availability and geographic closeness to the researcher. Subsequently, cluster sampling was utilized to select 20 intact administrative classes from these three universities, aiming to include at least one class of each major from each grade level in each university. The participants come from various provinces across China, with diverge regional backgrounds. Their majors cover a wide spectrum, including Primary Education, Financial Management, Chinese Language and Literature, Hotel Management, International Chinese Language Education, Network and New Media, Computer Science and Technology, Chemistry, Special Education, Preschool Education, and so on. The diversity of the participants' professional backgrounds not only enriches the sample composition but also enhances the generalization and applicability of the research results in similar educational scenarios. This enhances the explanatory power of the research findings among college EFL learners.

### 3.2 Data collection

Data were collected online through the “Questionnaire star” platform with QR code distributed via university course groups. The first page of the questionnaire presented a detailed informed consent form, stating the research purpose, emphasizing the principles of voluntary participation and information confidentiality, and clearly stating the right of participants to withdraw at any time. The questionnaire comprised five parts: demographic information, scales of perceived TES, BPNs satisfaction, FLE, and LE. All scales were presented in Chinese to ensure that participants fully understand. Clear instructions and sample items were provided to enhance response accuracy. To safeguard data quality, three attention-check items (e.g., “Please select ‘Strongly disagree' for this item”) were embedded within the questionnaire to identify and eliminate inattentive participants. All the submitted questionnaires that failed any of the attention-check items were excluded from the subsequent analysis. From a total of 512 submissions, 442 valid responses were retained after screening (32 for unreasonably short completion time, 11 for incomplete data, 27 for failure in attention-check items). The valid questionnaire rate reached 86%. The final valid sample consist of 73 boys and 369 girls, with an average age of 19. In terms of grade level, there were 248 (56%) freshmen and 194 (43%) sophomore.

### 3.3 Research instruments

#### 3.3.1 Teacher emotional support

This study measured the Perceptions of TES using the Teacher Emotional Support Scale validated by the Italian sample (Romano et al., 2020). This 15-item instrument comprises three subscales, as detailed below: The first is the positive climate subscale (with a total of 5 items, e.g., “Our teachers want students in this class to respect each other's ideas”). The second is the Teacher Sensitivity subscale (with a total of 6 items, e.g., “Our teacher cares about how we feel”). Third, the Regard for Adolescent Perspective subscale (with a total of 4 items, e.g., “Our teacher encourages us to help other students with their works”). Responses were captured on a 5-point Likert scale, where 1 represents “completely disagree” and 5 represents “strongly agree”. In this study, the scale demonstrated strong psychometric characteristics. The results of confirmatory factor analysis show an excellent model fit: χ^2^/ df = 2.353, GFI = 0.938, AGFI = 0.914, RMSEA = 0.055, CFI = 0.964. All indicators meet the recommended thresholds. Cronbach's alpha is 0.956, confirming the scale's high reliability.

#### 3.3.2 Basic psychological needs satisfaction

Students' BPNs satisfaction was assessed using a self-report questionnaire adapted from Zhou et al. (2023). The scale features three subscales, each measuring autonomy, competence, and relatedness, with three items in each subscale. The autonomy subscale assesses students' willingness to be autonomous and self-expression (e.g., “I feel free to be my 'true self' in this class”). The competence subscale reflects students' perceived effectiveness and control (e.g., “I do well in this class, even on the hard things”). The relatedness subscale evaluates students' sense of interpersonal connection (e.g., “I feel a strong sense of intimacy with people in this class”). All items were scored on a Likert scale, with higher scores indicating higher need satisfaction. This measurement tool has demonstrated satisfactory psychometric properties in prior educational research, including internal consistency and structural validity (e.g., Zhou et al., 2023). Confirmatory factor analysis (CFA) indicated excellent model fit: χ^2^/ df =1.054, GFI = 0.987, AGFI = 0.976, RMSEA = 0.011, CFI = 0.999, all meeting recommended thresholds. Cronbach's alpha was 0.840.

#### 3.3.3 Learning engagement

Students‘ learning engagement was evaluated using the multidimensional scale developed by Reeve and Tseng (2011), which assesses four distinct dimensions. Agentic engagement (5 items) captures students' proactive contributions to the learning process (e.g., “I offer suggestions about how to make the class better”). Behavioral engagement (5 items) reflects effort, attention, and concentration (e.g., “I listen carefully in class”). Emotional engagement (4 items) involves positive affective experiences (e.g., “Class is fun”). Cognitive engagement (8 items) indicates deep processing, self-regulation, and strategy application (e.g., “I try to relate what I'm learning to what I already know”). All items are rated on a Likert scale, with higher scores representing higher engagement. This scale has been widely used in educational research and has established robust reliability and validity. In this study, confirmatory factor analysis (CFA) demonstrated an excellent model fit: χ^2^/ df = 1.510, GFI = 0.948, AGFI = 0.933, RMSEA = 0.034, CFI = 0.984, all meeting recommended thresholds. Cronbach's alpha was 0.973, indicating robust reliability of the scale.

#### 3.3.4 Foreign language enjoyment

FLE was measured with the Chinese version of the Foreign Language Enjoyment Scale (Li et al., 2018), which has been previously validated in a Chinese high school setting. The 11-item scale assesses the extent of positive emotions experienced during the process of English learning. Sample items include “I enjoy it” and “It's a positive environment,” capturing facets such as personal enjoyment, pride, supportive atmosphere, and positive perceptions of teacher and peer support. Participants responded on a Likert scale from “Strongly Disagree” to “Strongly Agree”. The higher the score, the stronger enjoyment in the foreign language learning. The scale had shown good reliability and structural validity in studies involving Chinese EFL learners. Confirmatory factor analysis indicated an acceptable model fit: χ^2^/ df = 2.567, GFI = 0.935, AGFI = 0.912, RMSEA = 0.090, CFI = 0.939. Although the RMSEA value of 0.090 is at the upper threshold of acceptability, other indices such as CFI and GFI met the recommended criteria, supporting the use of the scale in this study. Cronbach's alpha was 0.900.

#### 3.3.5 Analytical procedures

Data were analyzed using SPSS 28.0 and AMOS 28.0 software. The analysis process mainly consisted of four steps. First, descriptive statistical analysis was conducted for all key variables (TES, BPNs, FLE and LE). Normality assumptions were first checked by assessing skewness and kurtosis. Pearson correlation analysis was then conducted to examine the bivariate relationships among the variables. The results showed that all values fell within the acceptable ranges, supporting the use of the maximum likelihood estimation method. The second step was to verify the effectiveness of the measurement scale. Confirmatory Factor Analysis (CFA) was performed separately for each construct to evaluate their factor structures and model fit. Following the validation of the measurement models, Structural Equation Modeling (SEM) based on maximum likelihood estimation was employed to test the hypothesized structural paths of the sequential mediation model. At the same time, both the direct effect of TES on LE and the indirect effects via BPNs satisfaction and FLE were evaluated. Finally, to rigorously evaluate the significance of the proposed mediation pathways, a bootstrap analysis with 5,000 resamples was conducted to generate bias-corrected confidence intervals for the indirect effects.

## 4 Results

### 4.1 Descriptive statistics and correlations

As summarized in Table 1, participants reported generally high levels on all measured constructs, with mean scores exceeding 4.0 on the response scales. Among them, TES received the highest mean score (*M* = 4.837, SD = 0.268), indicating that the participants perceived a relatively high level of TES. BPNs satisfaction exhibited the largest standard deviation (SD = 0.643), pointing to relatively greater individual differences in participants' experiences of need satisfaction.

**Table 1:** Descriptive statistics and correlation analysis.

**Variables**	**Min**	**Max**	**Skewness**	**Kurtosis**	**Mean**	**SD**	**TES**	**BPNs**	**FLE**	**LE**
TES	2.922	5	−2.467	8.089	4.837	0.268	1			
BPNs	1.667	5	−0.813	0.489	4.141	0.643	0.397^***^	1		
FLE	2.111	5	−0.832	0.494	4.254	0.566	0.396^***^	0.414^***^	1	
LE	2.167	5	−0.454	−0.12	4.014	0.564	0.450^***^	0.463^***^	0.468^***^	1

TES, teacher emotional support; BPNs, basic psychological needs satisfaction; LE learning engagement. *N* = 442. ^***^ *p* < 0.001.

Correlation analysis revealed that all variables were significantly and positively correlated with each other at the *p* < 0.001 level. Specifically, TES was significantly correlated with BPNs (*r* = 0.397), FLE (*r* = 0.396), and LE (*r* = 0.450). BPNs satisfaction was significantly correlated with FLE (*r* = 0.414) and LE (*r* = 0.463). Among them, the strongest bivariate correlation emerged between FLE and LE (*r* = 0.468).

These correlational patterns provide preliminary and favorable support for the proposed theoretical model, confirm the expected correlations among the constructs and establish a solid foundation for testing the sequential mediation hypothesis.

### 4.2 Mediation effect analysis

**Figure 2:**
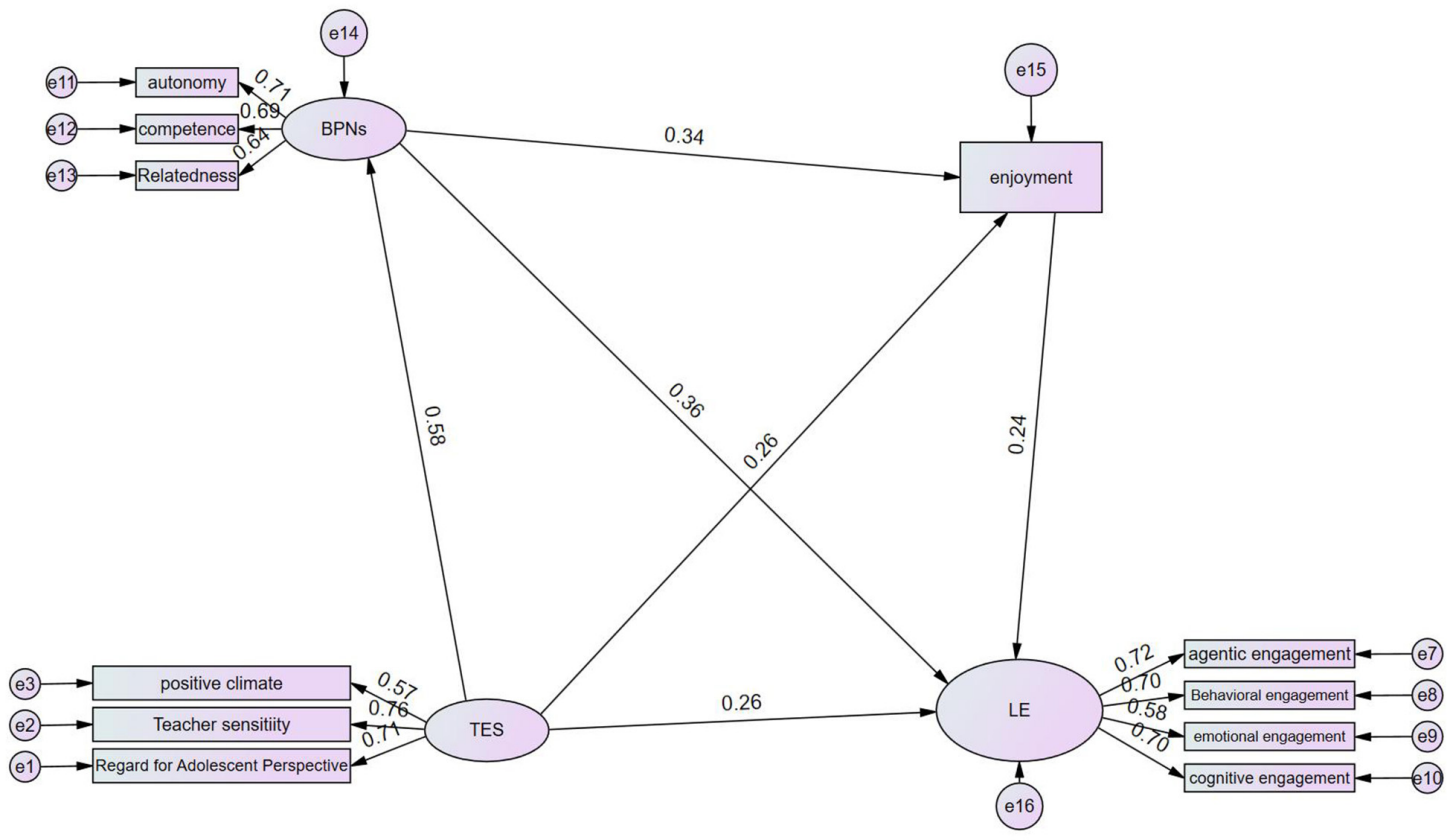
Path coefficients of the mediation model. *n* = 442. TES, teacher emotional support; BPNs, basic psychological needs satisfaction; LE, learning engagement.

The hypothesized sequential mediation model was tested using the structural equation model of AMOS 28.0. The results showed that the model fits well with the data: χ^2^/ df = 1.487, GFI = 0.977, CFI = 0.986, TLI = 0.980, RMSEA = 0.033, SRMR = 0.011(see Table 2), indicating that the theoretical model was well-supported empirically. The standardized path coefficients of the model are presented in Figure 2.

**Table 2:** Model fit index of the mediating role.

**Fit index**	***χ^2^/ df***	**GFI**	**RMR**	**RMSEA**	**CFI**	**NFI**	**TLI**	**AGFI**	**IFI**
Suggested value	< 3	>0.9	< 0.05	< 0.08	>0.9	>0.9	>0.9	>0.9	>0.9
Value of this study	1.487	0.977	0.011	0.033	0.986	0.959	0.980	0.961	0.986

Path analysis revealed a significant direct effect of TES on LE (β = 0.258, *p* < 0.001). The detailed path coefficients and their significance levels are shown in Table 3. In addition to this direct correlation, the analysis also revealed several significant indirect pathways, clarifying the intrinsic mechanisms through which TES influences LE.

**Table 3:** Path coefficients of the model.

**Path**	**Unstd. estimate (*B*)**	**S.E**.	**C.R**.	**Std. Estimate (β)**	***P***
TES → BPNs	1.247	0.165	7.567	0.581	< 0.001
BPNs → FLE	0.377	0.08	4.725	0.343	< 0.001
TES → FLE	0.603	0.165	3.657	0.255	< 0.001
FLE → LE	0.245	0.057	4.28	0.239	< 0.001
TES → LE	0.624	0.182	3.421	0.258	< 0.001
BPNs → LE	0.409	0.092	4.452	0.363	< 0.001

*n* = 442. TES, teacher emotional support; BPNs, basic psychological needs satisfaction; LE, Learning engagement.

First, the mediation effect analysis identified a significant indirect pathway through BPNs satisfaction. TES exerted a strong positive influence on BPNs satisfaction (β = 0.581, *p* < 0.001), which in turn significantly positively predicted LE (β = 0.343, *p* < 0.001). Bootstrap analysis confirmed that BPNs satisfaction served as a significant mediator in the relationship between TES and LE (indirect effect = 0.211, 95% CI [0.119, 0.358]) (see Table 4). This suggests that TES enhances LE partly by first fulfilling students' fundamental needs. Notably, this indirect effect accounted for 36.57% of the total effect, highlighting BPNs satisfaction as the primary psychological pathway linking TES to LE.

**Table 4:** Decomposition of the effects of TES on LE via BPNs and FLE.

**Paths**	**Stand. effect**	**95% CI**	**Effect proportion**
TES → BPNs → LE	0.211	[0.119, 0.358]	36.57%
TES → BPNs → FLE	0.199	[0.011, 0.334]	–
TES → FLE → LE	0.061	[0.019, 0.133]	10.57%
TES → BPNs → FLE → LE	0.048	[0.018, 0.099]	8.32%
The total indirect effect	0.319	[0.214, 0.47]	55.29%
The total effect	0.577	[0.446, 0.701]	

TES, teacher emotional support; BPNs, basic psychological needs satisfaction; LE, learning engagement.

Second, FLE was also found to be a significant mediator. Path analysis further revealed a significant direct effect from TES to FLE (β = 0.255, *p* < 0.001), and a similarly significant path from FLE to LE (β = 0.239, *p* < 0.001). The indirect effect of TES on LE through FLE alone was 0.061 (95% CI [0.019, 0.133]). This result confirms that TES also contributes to LE by enhancing students' FLE, which subsequently acts as an emotional driver of engaged learning behaviors.

Interestingly, the significant direct path from TES to FLE suggests that emotional support may also foster enjoyment through mechanisms beyond need satisfaction, such as direct emotional contagion or the creation of a positive classroom atmosphere. This finding adds nuance to the strict SDT sequence and suggests a more complex interplay between teacher support and student emotions.

In addition to these individual mediating paths, a significant sequential mediation pathway was identified. The path coefficients outline a clear motivational-emotional sequence: TES significantly positively predicted BPNs satisfaction (β = 0.581, *p* < .001), which subsequently fostered FLE (β = 0.343, *p* < 0.001), ultimately leading to enhanced LE (β = 0.239, *p* < 0.001). Bootstrap analysis confirmed that this specific indirect pathway was statistically significant (effect size = 0.048, 95% CI [0.018, 0.099]). This finding indicates that the satisfaction of basic psychological needs acts as a foundational resource that amplifies students' enjoyment in learning, which then translates into heightened engagement.

The complete decomposition of indirect effects, and total effects is provided in Table 4. Of note, while the direct effect of TES on LE (β = 0.258) remained significant, the total indirect effect (0.319) accounted for a substantial proportion of the total effect, highlighting the prominence of these motivational and emotional mechanisms in explaining how TES translates into students' LE.

## 5 Discussion

### 5.1 The direct effect of teacher emotional support on learning engagement

This study confirms that teacher emotional support positively predicts learning engagement among EFL learners, thereby verifying **H1**. This discovery further reinforces the well-established consensus that emotionally supportive teaching is an important cornerstone for students to engage in learning (Fredricks, 2011). As a key classroom contextual factor, teacher emotional support can make students feel cared, accepted and valued. This feeling can be directly translated into higher participation, manifested as enhanced vitality, focus, and dedication in the classroom (i.e., behavioral engagement), and strengthened identification, interest, and sense of value toward the school and class (i.e., emotional engagement) (Fredricks et al., 2019). This research extends previous findings by further refining this understanding: the direct effect, while significant, is notably smaller in magnitude than the total effect. This suggests that the influence of teacher emotional support is not simply a direct push, but is largely transmitted through internal psychological mechanisms (Roorda et al., 2011; Ryan and Deci, 2020).

### 5.2 The mediating effect of basic psychological needs satisfaction

This study identifies basic psychological needs satisfaction as the core mechanism underlying this relationship, confirming **H2**. This result aligns with existing SDT-based educational studies (e.g., Ahn et al., 2021; Zhou et al., 2022), and extends them into the EFL context with a more precise mechanistic account. From the SDT perspective (Ryan and Deci, 2000, 2020), this mediation effect can be understood as an organismic process: teacher emotional support, expressed through warmth, respect, and care, functions as a key social-contextual nutrient that fulfills students' innate psychological needs for autonomy, competence, and relatedness. Satisfying these needs, in turn, motivates students to invest more energy into learning.

Notably, our analysis shows that the indirect effect via basic psychological needs satisfaction is much stronger than other pathways. This highlights need fulfillment as the primary psychological route through which emotional support boosts engagement. Recent studies in both general education and EFL settings support this view. For instance, Xin (2022) found that perceived social support predicted Chinese undergraduates' engagement solely through needs satisfaction, accounting for the full indirect effect. Similarly, Bai and Gu (2024) observed that for Chinese K-12 online learners, teacher autonomy support and involvement enhanced engagement exclusively via the satisfaction of autonomy, competence, and relatedness needs, confirming the “support → needs → engagement” chain in digital environments. The primacy of needs satisfaction as a mediator is further corroborated by EFL-specific evidence. Dincer et al. (2019) reported that perceived teacher autonomy support predicted Turkish EFL learners‘ emotional and agentic engagement only indirectly through basic psychological needs satisfaction, with no significant direct effect remaining after accounting for the indirect path.

Notably, the mediating role of needs satisfaction appears robust across different educational levels and cultural contexts. Gao et al. (2021) found that among Chinese primary students, autonomy and relatedness satisfaction fully mediated the link between positive teacher-student relationships and emotional engagement, while competence satisfaction mediated the path to behavioral engagement. Buzzai et al. (2021) added nuance to this by identifying autonomy and relatedness—though not competence—as the primary drivers of academic engagement, implying that specific needs may contribute differently to engagement outcomes, a point worth exploring in future EFL research.

Overall, the consistency of these findings across diverse samples suggests that basic psychological needs satisfaction is more than a theoretical construct; it is a powerful mediator explaining how teacher emotional support leads to heightened engagement. By establishing needs satisfaction as the primary psychological pathway, our study refines the SDT framework and offers a clear implication for EFL pedagogy: fostering engagement starts with building emotionally supportive environments that meet students' fundamental needs.

### 5.3 The mediating effect of foreign language enjoyment

This study unveiled a modest mediation effect of enjoyment between teacher emotional support and learning engagement, confirming **H3**. Prior research has established that foreign language enjoyment serves as a significant mediator between teacher support and student engagement in EFL contexts (Zhao and Yang, 2022; Hosseini et al., 2022). This study confirmed this mediating role. However, we observed that the independent mediating effect of foreign language enjoyment (β = 0.061) was relatively modest compared to the other pathway in the model, which indicates that enjoyment serves as a valuable yet supplementary emotional catalyst rather than the primary driver. The significant mediating effect of enjoyment, though modest in magnitude, supports the core propositions of Control-Value Theory and broaden-and-build theory: teacher emotional support shapes the appraisals that elicit enjoyment, and this positive emotion, in turn, sustains engagement (Pekrun, 2024; Fredrickson, 2001).

Upon conducting a comprehensive analysis of the sequential mediation model, we determined that while teacher emotional support directly predicted foreign language enjoyment (β = 0.255, *p* < 0.001), a substantial portion of its influence on enjoyment was actually transmitted through the prior fulfillment of basic psychological needs (TES → BPNs → FLE, β = 0.199). This finding aligns with the theoretical premise that need satisfaction creates the psychological foundation upon which positive achievement emotions can flourish (Ryan and Deci, 2020; Pekrun, 2024). This suggests that teacher emotional support requires the foundational mediation of psychological need satisfaction to fully manifest in students' sustained foreign language enjoyment. While enjoyable experiences provide immediate emotional boosts, their sustained impact on engagement is enhanced when grounded in solid psychological need fulfillment.

The relatively modest independent mediation effect of FLE might be largely attributed to the nature of academic emotions and the specific cultural context. Regarding the nature of emotions, enjoyment as a positive affect tends to be more transient and situation-specific compared to the more enduring psychological state of need satisfaction, which may limit its sustained impact as an independent mediator. From a cultural perspective, Chinese EFL learners' engagement might be more strongly driven by deep-seated motivational factors than by immediate emotional experiences. In Confucian heritage cultures, sustained academic effort often stems from a sense of responsibility and long-term goals rather than momentary positive affect (Lin and Chen, 2024; Zhou et al., 2012). For example, Confucian heritage cultures value relational orientation, which suggests that students' perceptions of teacher emotional support are not merely interpreted as sources of immediate enjoyment, but are often internalized as a sense of interpersonal obligation and gratitude. When teachers provide support, it may activate a strong desire to meet the teacher's expectations, thereby strengthening the path from TES to BPNs satisfaction, particularly the need for relatedness. Consequently, the motivational impetus for engagement flows more directly from this deep-seated sense of relational responsibility than from the transient affect of enjoyment.

### 5.4 The sequential mediating effect of basic psychological needs satisfaction and foreign language enjoyment

This study empirically validates a significant sequential mediation pathway, confirming **H4**. While its effect size (β = 0.048) was smaller than the independent mediating effect of basic needs satisfaction (β = 0.211), this finding holds important theoretical value by integrating and deepening our understanding of the motivational-affective processes underlying teacher support.

This sequential pathway demonstrates that the internalization of teacher support can follow a dynamic process where contextual support first nurtures students' innate needs for autonomy, competence, and relatedness. This enhanced motivational foundation then creates fertile ground for the growth of positive academic emotions like enjoyment, which subsequently act as a proximal emotional driver for engaged learning behaviors.

This finding represents a significant theoretical advancement by providing strong empirical support for the motivational—emotional sequence that has long postulated by SDT (Ryan and Deci, 2020) but rarely tested in full within EFL research. The results of this study crucially extend previous studies that only examined this sequence fragment in isolation. While Zhou et al. (2022) demonstrated the the link between BPNs satisfaction and LE, Zhao and Yang (2022) established the mediation path of enjoyment, our study is among the first studies in the EFL domain to empirically integrate these mechanisms into a complete “TES → BPNs → FLE → LE” sequence. This framework resolves theoretical ambiguity by clearly positioning BPNs satisfaction as an antecedent to FLE, rather than treating them as parallel mediators. Our findings align with and extend the recent experimental evidence from Alrabai and Algazzaz (2024) by demonstrating this chain for a multidimensional LE construct, thereby offering broader validation and a more comprehensive explanatory framework.

At the theoretical level, this sequential mediation clearly outlines the precise psychological micro-process through which TES gets internalized. It uncovers the “black box” between contextual support and engaged behavior, revealing a dynamic process where supportive teaching first satisfies students' BPNs, then stimulates the positive emotional experience of enjoyment, and ultimately externalizes it into observable engagement behaviors.

At the practical level, this refined understanding provides critical insights for pedagogical intervention: efforts to enhance engagement must simultaneously take into account both the motivational and affective dimensions. Interventions structured to first bolster students' sense of autonomy, competence, and relatedness may be particularly effective in creating the conditions for genuine enjoyment and, consequently promoting their formation of continuous learning commitment. This underscores the importance of comprehensive teacher training programs that equip educators with strategies to nurture both the psychological wellbeing and emotional experiences of their students.

### 5.5 Research limitations and future directions

This study, by validating a sequential mediation model linking teacher emotional support to learning engagement through basic psychological needs satisfaction and foreign language enjoyment, provides a nuanced understanding of the motivational-affective mechanisms operating in the Chinese EFL classroom. This integrated framework not only advances theoretical knowledge by bridging SDT theory with Control-Value Theory in EFL but also offers empirically-grounded insights for developing more effective pedagogical practices. While these contributions are significant, several limitations inherent in this investigation should be acknowledged.

First, the cross-sectional nature of our data, while suitable for establishing correlational relationships and testing the mediation model, inherently precludes definitive causal inferences. Future studies should therefore prioritize longitudinal or experimental designs to corroborate the causal directions proposed in our sequential model. Second, despite using validated instruments, our reliance on self-reported data may introduce the risk of common method bias. To triangulate findings and enhance the robustness of the evidence, future research would benefit greatly from a multi-method, multi-informant approach. Third, the generalizability of our findings might be constrained by the specific participant profile. The sample was exclusively composed of non-English majors from universities in Hainan province, which, while diverse in academic disciplines, represents a particular segment of the broader EFL learner population. It is also important to note that the sample was predominantly female (83.5%). Given that students of different genders may differ in their perceived teacher support (e.g., Liu and Li, 2023), basic psychological needs satisfaction and enjoyment (Feng et al., 2023), the current results should be generalized to male EFL learners with caution. However, some studies (e.g., Ma and Li, 2026) reported that gender did not significantly moderate the paths from teacher emotional support to academic engagement. Future studies with a more balanced gender distribution are needed to verify the model's applicability across genders.

## 6 Conclusion

Based Mainly on Self-Determination Theory, we constructed a sequential mediation model to explore the psychological mechanisms linking teacher emotional support to EFL learning engagement. We found that teacher emotional support enhances EFL students' learning engagement through multiple pathways. The most potent of these is the satisfaction of basic psychological needs. Foreign language enjoyment also serves as a significant mediator. Furthermore, a sequential chain wherein need satisfaction fosters enjoyment, which in turn drives engagement, was empirically supported, offering a comprehensive motivational-affective framework for understanding the impact of teacher emotional support.

## Data Availability

The original contributions presented in the study are included in the article/Supplementary material, further inquiries can be directed to the corresponding author.
